# *trans*-Dichloridobis[diphen­yl(4-vinyl­phen­yl)phosphane-κ*P*]palladium(II)

**DOI:** 10.1107/S1600536811044795

**Published:** 2011-11-05

**Authors:** Reinout Meijboom

**Affiliations:** a Research Centre for Synthesis and Catalysis, Department of Chemistry, University of Johannesburg, PO Box 524, Auckland Park 2006, Johannesburg, South Africa

## Abstract

In the title compound, [PdCl_2_(C_20_H_17_P)_2_], the Pd^II^ atom lies on a center of symmetry, resulting in a distorted *trans*-square-planar geometry. The Pd—P and Pd—Cl bond lengths are 2.3366 (7) and 2.2966 (7) Å, respectively. The vinyl group is disordered over two sets of sites in a 0.696 (15):0.304 (15) ratio.

## Related literature

For a review on related compounds, see: Spessard & Miessler (1996 ▶). For the synthesis of the starting materials, see: Drew & Doyle (1990 ▶). For similar *R*-P_2_PdCl_2_ compounds, see: Ogutu & Meijboom (2011 ▶); Muller & Meijboom (2010*a* ▶,*b* ▶).
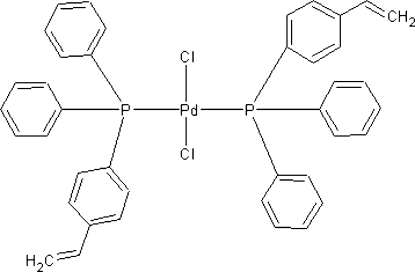


## Experimental

### 

#### Crystal data

[PdCl_2_(C_20_H_17_P)_2_]
*M* *_r_* = 753.91
Triclinic, 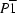

*a* = 9.9495 (3) Å
*b* = 9.9512 (3) Å
*c* = 10.4387 (4) Å
α = 67.683 (2)°
β = 86.366 (2)°
γ = 61.979 (2)°
*V* = 835.62 (5) Å^3^
*Z* = 1
Mo *K*α radiation
μ = 0.84 mm^−1^
*T* = 100 K
0.24 × 0.18 × 0.06 mm

#### Data collection

Bruker APEXII CCD diffractometer
Absorption correction: multi-scan (*SADABS*; Bruker, 2004 ▶) *T*_min_ = 0.837, *T*_max_ = 0.955
5829 measured reflections
2723 independent reflections
2682 reflections with *I* > 2σ(*I*)
*R*_int_ = 0.030

#### Refinement

*R*[*F* ^2^ > 2σ(*F* ^2^)] = 0.041
*wR*(*F* ^2^) = 0.105
*S* = 1.07
2723 reflections
205 parameters
H-atom parameters constrained
Δρ_max_ = 1.66 e Å^−3^
Δρ_min_ = −0.75 e Å^−3^

### 

Data collection: *APEX2* (Bruker, 2005 ▶); cell refinement: *SAINT-Plus* (Bruker, 2004 ▶); data reduction: *SAINT-Plus* and *XPREP* (Bruker, 2004 ▶); program(s) used to solve structure: *SHELXS97* (Sheldrick, 2008 ▶); program(s) used to refine structure: *SHELXL97* (Sheldrick, 2008 ▶); molecular graphics: *DIAMOND* (Brandenburg, 2005 ▶); software used to prepare material for publication: *WinGX* (Farrugia, 1999 ▶).

## Supplementary Material

Crystal structure: contains datablock(s) global, I. DOI: 10.1107/S1600536811044795/fi2114sup1.cif

Structure factors: contains datablock(s) I. DOI: 10.1107/S1600536811044795/fi2114Isup2.hkl

Additional supplementary materials:  crystallographic information; 3D view; checkCIF report

## Figures and Tables

**Fig. 1.**
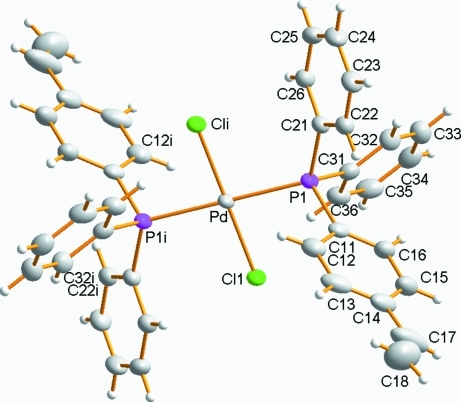
The structure of the title compound, showing 50% probability displacement ellipsoids. For the C atoms, the first digit indicates ring number and the second digit indicates the position of the atom in the ring. Some labels have been omitted for clarity, all rings have been numbered in the same, systematic manner. H atoms are depicted by arbitrary size spheres. Primed atoms are generated by i: –x, –y, 1 – z.

## supplementary crystallographic information

### Comment

Transition metal complexes containing phosphine, arsine and stibine ligands are widely being investigated in various fields of organometallic chemistry (Spessard & Miessler, 1996). As part of a systematic investigation involving complexes with the general formula *trans*-[*MX*_2_(*L*)_2_] (*M* = Pt or Pd; *X* = halogen, Me, Ph; *L* = Group 15 donor ligand), crystals of the title compound, were obtained.

[PdCl_2_(*L*)_2_] (*L* = tertiary phosphine, arsine or stibine) complexes can conveniently be prepared by the substitution of 1,5-cyclooctadiene (COD) from [PdCl_2_(COD)]. The title compound, *trans*-[PdCl_2_{P(4—H_2_C=CHC_6_H_4_)Ph_2_}_2_], crystallizes in the triclinic spacegroup *P*1, with the Pd atom on a center of symmetry and each pair of equivalent ligands in a mutually *trans* orientation. The geometry is, therefore, slightly distorted square planar and the Pd atom is not elevated out of the coordinating atom plane. All angles in the coordination polyhedron are close to the ideal value of 90°, with P—Pd—Cl = 85.83 (4)° and P—Pd—Cl^i^ = 94.17 (4)°. As required by the crystallographic symmetry, the P—Pd—P^i^ and Cl—Pd—Cl^i^ angles are 180°. No weak intermolecular interactions were observed.

The title compound compares well with other closely related Pd^II^ complexes from the literature containing two chloro and two tertiary phosphine ligands in a *trans* geometry (Muller & Meijboom, 2010*a*; 2010*b*). The title compound, having a Pd—Cl bond length of 2.2969 (14) Å and a Pd—P bond length of 2.3367 (12) Å, fits well into the typical range for complexes of this kind. Notably the title compound did not crystallize as a solvated complex; these type of Pd^II^ complexes have a tendency to crystallize as solvates (Meijboom & Omondi, 2011).

Large thermal vibrations on the periphery of the molecule results in a poorly defined C=C bond length. Disordered modelling resulted in an unstable refinement.

### Experimental

Diphenylphosphinostyrene (0.05 g, 0.35 mmol) was dissolved in acetone (5 cm^3^). A solution of [Pd(COD)Cl_2_] (0.05 g, 0.17 mmol) in acetone (5 cm^3^) was added to the phosphine solution. The mixture was stirred for 5 minutes, after which the solution was left to crystallize. Yellow crystals of the title compound were obtained.

### Refinement

H atoms were placed in geometrically idealized positions (C—H = 0.95–0.98) and constrained to ride on their parent atoms with *U*_iso_(H) = 1.2*U*_eq_(C).

### Crystal data

**Table:**

[PdCl_2_(C_20_H_17_P)_2_]	*Z* = 1
*M**_r_* = 753.91	*F*(000) = 384
Triclinic, *P*1	*D*_x_ = 1.498 Mg m^−^^3^
Hall symbol: -P 1	Mo *K*α radiation, λ = 0.71069 Å
*a* = 9.9495 (3) Å	Cell parameters from 5189 reflections
*b* = 9.9512 (3) Å	θ = 4.6–64.1°
*c* = 10.4387 (4) Å	µ = 0.84 mm^−^^1^
α = 67.683 (2)°	*T* = 100 K
β = 86.366 (2)°	Plate, yellow
γ = 61.979 (2)°	0.24 × 0.18 × 0.06 mm
*V* = 835.62 (5) Å^3^	

### Data collection

**Table:**

Bruker APEXII CCD diffractometer	2723 independent reflections
Radiation source: sealed tube	2682 reflections with *I* > 2σ(*I*)
graphite	*R*_int_ = 0.030
φ and ω scans	θ_max_ = 24.6°, θ_min_ = 2.1°
Absorption correction: multi-scan (*SADABS*; Bruker, 2004)	*h* = −11→8
*T*_min_ = 0.837, *T*_max_ = 0.955	*k* = −11→9
5829 measured reflections	*l* = −11→12

### Refinement

**Table:**

Refinement on *F*^2^	Primary atom site location: structure-invariant direct methods
Least-squares matrix: full	Secondary atom site location: difference Fourier map
*R*[*F*^2^ > 2σ(*F*^2^)] = 0.041	Hydrogen site location: inferred from neighbouring sites
*wR*(*F*^2^) = 0.105	H-atom parameters constrained
*S* = 1.07	*w* = 1/[σ^2^(*F*_o_^2^) + (0.0461*P*)^2^ + 2.1233*P*] where *P* = (*F*_o_^2^ + 2*F*_c_^2^)/3
2723 reflections	(Δ/σ)_max_ < 0.001
205 parameters	Δρ_max_ = 1.66 e Å^−^^3^
0 restraints	Δρ_min_ = −0.75 e Å^−^^3^

### Special details

**Table:**

Geometry. All e.s.d.'s (except the e.s.d. in the dihedral angle between two l.s. planes) are estimated using the full covariance matrix. The cell e.s.d.'s are taken into account individually in the estimation of e.s.d.'s in distances, angles and torsion angles; correlations between e.s.d.'s in cell parameters are only used when they are defined by crystal symmetry. An approximate (isotropic) treatment of cell e.s.d.'s is used for estimating e.s.d.'s involving l.s. planes.
Refinement. Refinement of *F*^2^ against ALL reflections. The weighted *R*-factor *wR* and goodness of fit *S* are based on *F*^2^, conventional *R*-factors *R* are based on *F*, with *F* set to zero for negative *F*^2^. The threshold expression of *F*^2^ > σ(*F*^2^) is used only for calculating *R*-factors(gt) *etc*. and is not relevant to the choice of reflections for refinement. *R*-factors based on *F*^2^ are statistically about twice as large as those based on *F*, and *R*- factors based on ALL data will be even larger.

### Fractional atomic coordinates and isotropic or equivalent isotropic displacement parameters (Å^2^)

**Table:**

	*x*	*y*	*z*	*U*_iso_*/*U*_eq_	Occ. (<1)
C11	0.8766 (3)	0.2614 (4)	0.6548 (3)	0.0283 (6)	
C12	0.8919 (4)	0.1305 (4)	0.6246 (3)	0.0364 (7)	
H12	0.96	0.0217	0.683	0.044*	
C13	0.8047 (4)	0.1631 (5)	0.5062 (4)	0.0441 (9)	
H13	0.8175	0.0753	0.4853	0.053*	
C14	0.6994 (4)	0.3238 (5)	0.4194 (3)	0.0468 (9)	
C15	0.6826 (4)	0.4504 (5)	0.4536 (3)	0.0459 (9)	
H15	0.6107	0.5589	0.3981	0.055*	
C16	0.7708 (4)	0.4203 (4)	0.5696 (3)	0.0355 (7)	
H16	0.7579	0.5087	0.5897	0.043*	
C17	0.5932 (8)	0.3815 (11)	0.2904 (8)	0.0354 (17)	0.696 (15)
H17	0.5121	0.4896	0.2551	0.042*	0.696 (15)
C18	0.6107 (6)	0.2857 (8)	0.2271 (6)	0.046 (2)	0.696 (15)
H18A	0.6911	0.1771	0.2609	0.056*	0.696 (15)
H18B	0.543	0.3255	0.148	0.056*	0.696 (15)
C19	0.6584 (19)	0.292 (2)	0.3018 (15)	0.034 (4)	0.304 (15)
H19	0.6993	0.1868	0.3024	0.04*	0.304 (15)
C20	0.5616 (16)	0.424 (2)	0.200 (2)	0.050 (5)	0.304 (15)
H20A	0.5235	0.5275	0.2037	0.06*	0.304 (15)
H20B	0.5298	0.416	0.1226	0.06*	0.304 (15)
C21	1.1852 (3)	0.1752 (4)	0.7390 (3)	0.0278 (6)	
C22	1.2089 (3)	0.1773 (4)	0.6054 (3)	0.0312 (6)	
H22	1.1285	0.2002	0.5456	0.037*	
C23	1.3508 (4)	0.1456 (4)	0.5606 (3)	0.0364 (7)	
H23	1.3655	0.147	0.4713	0.044*	
C24	1.4702 (4)	0.1119 (4)	0.6490 (4)	0.0414 (8)	
H24	1.5659	0.0893	0.6197	0.05*	
C25	1.4476 (4)	0.1118 (4)	0.7813 (3)	0.0380 (7)	
H25	1.5278	0.0907	0.8401	0.046*	
C26	1.3067 (3)	0.1429 (4)	0.8262 (3)	0.0325 (6)	
H26	1.2926	0.1423	0.9153	0.039*	
C31	0.9371 (3)	0.4102 (4)	0.8217 (3)	0.0282 (6)	
C32	0.9929 (4)	0.5164 (4)	0.7448 (3)	0.0331 (7)	
H32	1.063	0.4878	0.6839	0.04*	
C33	0.9444 (4)	0.6660 (4)	0.7584 (3)	0.0379 (7)	
H33	0.9824	0.7368	0.707	0.045*	
C34	0.8397 (4)	0.7090 (4)	0.8483 (3)	0.0376 (7)	
H34	0.8065	0.8091	0.857	0.045*	
C35	0.7847 (4)	0.6031 (4)	0.9250 (4)	0.0404 (8)	
H35	0.7141	0.6326	0.9852	0.048*	
C36	0.8330 (3)	0.4529 (4)	0.9140 (3)	0.0349 (7)	
H36	0.7965	0.3814	0.9674	0.042*	
P1	1.00121 (8)	0.21439 (9)	0.80338 (7)	0.02397 (18)	
Cl1	0.73685 (8)	0.14865 (9)	0.94644 (8)	0.03426 (19)	
Pd	1	0	1	0.02362 (13)	

### Atomic displacement parameters (Å^2^)

**Table:**

	*U*^11^	*U*^22^	*U*^33^	*U*^12^	*U*^13^	*U*^23^
C11	0.0233 (14)	0.0424 (16)	0.0255 (14)	−0.0213 (13)	0.0092 (11)	−0.0135 (13)
C12	0.0306 (16)	0.0466 (18)	0.0424 (17)	−0.0231 (15)	0.0150 (13)	−0.0234 (15)
C13	0.0453 (19)	0.077 (3)	0.048 (2)	−0.046 (2)	0.0287 (16)	−0.044 (2)
C14	0.050 (2)	0.083 (3)	0.0261 (16)	−0.052 (2)	0.0107 (15)	−0.0155 (17)
C15	0.047 (2)	0.060 (2)	0.0327 (17)	−0.0375 (18)	−0.0034 (15)	−0.0030 (16)
C16	0.0333 (16)	0.0426 (17)	0.0327 (16)	−0.0243 (15)	0.0012 (13)	−0.0088 (14)
C17	0.029 (3)	0.041 (4)	0.033 (4)	−0.016 (3)	0.004 (3)	−0.013 (3)
C18	0.047 (3)	0.050 (4)	0.038 (3)	−0.020 (3)	−0.006 (2)	−0.015 (3)
C19	0.031 (8)	0.039 (8)	0.036 (8)	−0.016 (7)	0.006 (6)	−0.022 (6)
C20	0.052 (8)	0.064 (11)	0.030 (9)	−0.026 (7)	−0.002 (6)	−0.016 (8)
C21	0.0251 (14)	0.0305 (14)	0.0279 (14)	−0.0158 (12)	0.0084 (11)	−0.0094 (12)
C22	0.0293 (15)	0.0342 (15)	0.0303 (15)	−0.0178 (13)	0.0053 (12)	−0.0099 (12)
C23	0.0334 (17)	0.0416 (17)	0.0333 (16)	−0.0195 (14)	0.0155 (13)	−0.0139 (14)
C24	0.0271 (16)	0.0444 (18)	0.049 (2)	−0.0192 (15)	0.0156 (14)	−0.0136 (16)
C25	0.0285 (16)	0.0414 (17)	0.0406 (18)	−0.0198 (14)	0.0000 (13)	−0.0083 (14)
C26	0.0316 (16)	0.0376 (16)	0.0283 (15)	−0.0197 (13)	0.0054 (12)	−0.0095 (13)
C31	0.0245 (14)	0.0349 (15)	0.0241 (14)	−0.0131 (12)	0.0018 (11)	−0.0119 (12)
C32	0.0373 (16)	0.0405 (17)	0.0244 (14)	−0.0201 (14)	0.0083 (12)	−0.0144 (13)
C33	0.0447 (18)	0.0395 (17)	0.0318 (16)	−0.0231 (15)	0.0062 (14)	−0.0129 (14)
C34	0.0360 (17)	0.0398 (17)	0.0356 (17)	−0.0136 (14)	−0.0012 (13)	−0.0186 (14)
C35	0.0307 (16)	0.056 (2)	0.0425 (18)	−0.0186 (15)	0.0106 (14)	−0.0306 (16)
C36	0.0314 (16)	0.0473 (18)	0.0334 (16)	−0.0226 (14)	0.0095 (12)	−0.0194 (14)
P1	0.0213 (4)	0.0307 (4)	0.0205 (3)	−0.0149 (3)	0.0058 (3)	−0.0081 (3)
Cl1	0.0207 (3)	0.0380 (4)	0.0347 (4)	−0.0152 (3)	0.0042 (3)	−0.0039 (3)
Pd	0.01846 (18)	0.02981 (19)	0.02028 (18)	−0.01383 (14)	0.00523 (11)	−0.00526 (13)

### Geometric parameters (Å, °)

**Table:**

C11—C16	1.373 (4)	C22—H22	0.93
C11—C12	1.391 (4)	C23—C24	1.380 (5)
C11—P1	1.820 (3)	C23—H23	0.93
C12—C13	1.397 (5)	C24—C25	1.385 (5)
C12—H12	0.93	C24—H24	0.93
C13—C14	1.387 (6)	C25—C26	1.378 (4)
C13—H13	0.93	C25—H25	0.93
C14—C15	1.371 (5)	C26—H26	0.93
C14—C19	1.498 (14)	C31—C32	1.385 (4)
C14—C17	1.511 (8)	C31—C36	1.398 (4)
C15—C16	1.392 (4)	C31—P1	1.827 (3)
C15—H15	0.93	C32—C33	1.395 (4)
C16—H16	0.93	C32—H32	0.93
C17—C18	1.298 (12)	C33—C34	1.382 (5)
C17—H17	0.93	C33—H33	0.93
C18—H18A	0.93	C34—C35	1.377 (5)
C18—H18B	0.93	C34—H34	0.93
C19—C20	1.29 (3)	C35—C36	1.388 (5)
C19—H19	0.93	C35—H35	0.93
C20—H20A	0.93	C36—H36	0.93
C20—H20B	0.93	P1—Pd	2.3366 (7)
C21—C22	1.393 (4)	Cl1—Pd	2.2966 (7)
C21—C26	1.395 (4)	Pd—Cl1^i^	2.2966 (7)
C21—P1	1.824 (3)	Pd—P1^i^	2.3366 (7)
C22—C23	1.386 (4)		
			
C16—C11—C12	118.8 (3)	C22—C23—H23	120.1
C16—C11—P1	122.8 (2)	C23—C24—C25	120.1 (3)
C12—C11—P1	118.4 (2)	C23—C24—H24	120
C11—C12—C13	119.8 (3)	C25—C24—H24	120
C11—C12—H12	120.1	C26—C25—C24	120.3 (3)
C13—C12—H12	120.1	C26—C25—H25	119.9
C14—C13—C12	121.3 (3)	C24—C25—H25	119.9
C14—C13—H13	119.4	C25—C26—C21	120.5 (3)
C12—C13—H13	119.4	C25—C26—H26	119.8
C15—C14—C13	117.9 (3)	C21—C26—H26	119.8
C15—C14—C19	141.5 (8)	C32—C31—C36	119.7 (3)
C13—C14—C19	99.8 (8)	C32—C31—P1	120.0 (2)
C15—C14—C17	113.6 (5)	C36—C31—P1	120.3 (2)
C13—C14—C17	128.5 (4)	C31—C32—C33	120.2 (3)
C14—C15—C16	121.5 (3)	C31—C32—H32	119.9
C14—C15—H15	119.3	C33—C32—H32	119.9
C16—C15—H15	119.3	C34—C33—C32	120.0 (3)
C11—C16—C15	120.7 (3)	C34—C33—H33	120
C11—C16—H16	119.6	C32—C33—H33	120
C15—C16—H16	119.6	C35—C34—C33	119.8 (3)
C18—C17—C14	122.4 (7)	C35—C34—H34	120.1
C18—C17—H17	118.8	C33—C34—H34	120.1
C14—C17—H17	118.8	C34—C35—C36	121.1 (3)
C17—C18—H18A	120	C34—C35—H35	119.5
C17—C18—H18B	120	C36—C35—H35	119.5
H18A—C18—H18B	120	C35—C36—C31	119.3 (3)
C20—C19—C14	114.3 (15)	C35—C36—H36	120.4
C20—C19—H19	122.8	C31—C36—H36	120.4
C14—C19—H19	122.8	C11—P1—C21	103.88 (13)
C19—C20—H20A	120	C11—P1—C31	106.35 (13)
C19—C20—H20B	120	C21—P1—C31	102.82 (13)
H20A—C20—H20B	120	C11—P1—Pd	110.15 (9)
C22—C21—C26	118.6 (3)	C21—P1—Pd	116.81 (10)
C22—C21—P1	122.5 (2)	C31—P1—Pd	115.66 (9)
C26—C21—P1	118.9 (2)	Cl1—Pd—Cl1^i^	180
C23—C22—C21	120.8 (3)	Cl1—Pd—P1	85.83 (2)
C23—C22—H22	119.6	Cl1^i^—Pd—P1	94.17 (2)
C21—C22—H22	119.6	Cl1—Pd—P1^i^	94.17 (2)
C24—C23—C22	119.7 (3)	Cl1^i^—Pd—P1^i^	85.83 (2)
C24—C23—H23	120.1	P1—Pd—P1^i^	180
			
C16—C11—C12—C13	2.5 (4)	C32—C33—C34—C35	0.5 (5)
P1—C11—C12—C13	−176.1 (2)	C33—C34—C35—C36	0.2 (5)
C11—C12—C13—C14	−1.7 (4)	C34—C35—C36—C31	−1.1 (5)
C12—C13—C14—C15	−0.4 (5)	C32—C31—C36—C35	1.3 (4)
C12—C13—C14—C19	171.6 (5)	P1—C31—C36—C35	−179.3 (2)
C12—C13—C14—C17	−178.2 (4)	C16—C11—P1—C21	−101.2 (3)
C13—C14—C15—C16	1.6 (5)	C12—C11—P1—C21	77.3 (2)
C19—C14—C15—C16	−165.6 (9)	C16—C11—P1—C31	6.9 (3)
C17—C14—C15—C16	179.8 (4)	C12—C11—P1—C31	−174.6 (2)
C12—C11—C16—C15	−1.3 (4)	C16—C11—P1—Pd	132.9 (2)
P1—C11—C16—C15	177.2 (2)	C12—C11—P1—Pd	−48.6 (2)
C14—C15—C16—C11	−0.8 (5)	C22—C21—P1—C11	−2.7 (3)
C15—C14—C17—C18	167.1 (5)	C26—C21—P1—C11	176.8 (2)
C13—C14—C17—C18	−15.0 (7)	C22—C21—P1—C31	−113.5 (3)
C19—C14—C17—C18	5.3 (9)	C26—C21—P1—C31	66.1 (3)
C15—C14—C19—C20	−9.9 (16)	C22—C21—P1—Pd	118.8 (2)
C13—C14—C19—C20	−178.5 (10)	C26—C21—P1—Pd	−61.7 (3)
C17—C14—C19—C20	17.6 (9)	C32—C31—P1—C11	−85.8 (2)
C26—C21—C22—C23	0.9 (4)	C36—C31—P1—C11	94.9 (2)
P1—C21—C22—C23	−179.5 (2)	C32—C31—P1—C21	23.1 (3)
C21—C22—C23—C24	−0.2 (5)	C36—C31—P1—C21	−156.3 (2)
C22—C23—C24—C25	−0.8 (5)	C32—C31—P1—Pd	151.6 (2)
C23—C24—C25—C26	0.9 (5)	C36—C31—P1—Pd	−27.8 (3)
C24—C25—C26—C21	−0.2 (5)	C11—P1—Pd—Cl1	−48.46 (10)
C22—C21—C26—C25	−0.7 (4)	C21—P1—Pd—Cl1	−166.62 (11)
P1—C21—C26—C25	179.7 (2)	C31—P1—Pd—Cl1	72.14 (10)
C36—C31—C32—C33	−0.6 (4)	C11—P1—Pd—Cl1^i^	131.54 (10)
P1—C31—C32—C33	180.0 (2)	C21—P1—Pd—Cl1^i^	13.38 (11)
C31—C32—C33—C34	−0.3 (5)	C31—P1—Pd—Cl1^i^	−107.86 (10)

Symmetry codes: (i) −*x*+2, −*y*, −*z*+2.
